# Interrogating pulmonary diffusing capacity in long COVID: insights from DLCO and DLNO testing

**DOI:** 10.3389/fphys.2025.1725263

**Published:** 2025-11-24

**Authors:** Jordan K. Parks, Bruce D. Johnson, Meredith G. Shea, Chul-Ho Kim, Jessica I. Johnston, Alex Carlson, Jesse C. Schwartz, Courtney M. Wheatley-Guy

**Affiliations:** 1 Mayo Clinic, Division of Cardiovascular Diseases, Scottsdale, AZ, United States; 2 Mayo Clinic, Division of Cardiovascular Diseases, Rochester, MN, United States

**Keywords:** long COVID, COVID, diffusion capacities for CO and NO, alveolar volume, membrane conductance

## Abstract

**Introduction:**

The lingering respiratory effects of COVID-19, particularly in patients with Long COVID, remain poorly understood, prompting a comprehensive evaluation of lung function in this population.

**Methods:**

Simultaneous measurements of diffusion capacity of the lungs for carbon monoxide (DLCO) and nitric oxide (DLNO), chest computed tomography (CT), lung ultrasound and questionnaires were collected in 74 subjects. Participants were categorized into two groups: those that have no lingering symptoms (NS, n = 37) and those still struggling with symptoms after initial infection, the disease known as Long COVID (LC, n = 37).

**Results:**

DLCO and DLNO were significantly lower in the LC group compared to the NS group (LC vs. NS, DLCO: 25.94 ± 7.65 vs. 21.71 ± 6.35 mL/min/mmHg, p = 0.009; DLNO: 148.5 ± 35.6 vs. 126.6 ± 32.2 mL/min/mmHg, p = 0.006). Pulmonary capillary blood volume (Vc) was also significantly lower in the LC group (43.38 ± 13.87, 70.79 ± 17.77, p = 0.003; LC vs. NS, respectively). Alveolar volume (VA) is significantly lower in the LC group (LC vs. NS, 5.06 ± 1.17 vs. 5.95 ± 1.16, p = 0.004). There was no significant difference between groups for surface area of the lungs available for gas exchange by resistance to gas transfer across the alveolar-capillary membrane (DM) between groups (LC vs. NS, 208.63 ± 97.3, 223.0 ± 93.47 mL/min/mmHg, p = 0.54). These findings indicate that Vc is the driving factor of decreased DLCO. CT findings and lung ultrasound showed no differences between the two groups for lung fluid (p = 0.525; p = 0.298).

**Conclusion:**

These findings suggest that a lack of volume available for perfusion could be problematic for these patients and as such requires further investigation for clinical management of these patients.

## 1 Introduction

The complications that follow a COVID-19 (SARS-CoV-2) infection can range from mild to life debilitating without a seemingly obvious reason. The people inflicted with these persistent symptoms are diagnosed with Long COVID (LC). These people with LC deal with symptoms including shortness of breath, brain fog and decreased exercise capacity (Nugent and Berdine, 2024; Durstenfeld et al., 2023). This study aimed to understand and explain the differences between people with LC and those without by utilizing non-invasive pulmonary testing including typical pulmonary function measures, such as forced vital capacity (FVC), slow vital capacity (SVC), and measuring the diffusion capacity of the lungs for carbon monoxide (DLCO) and nitric oxide (DLNO). These measurements have been recorded previously in the acute infection stage as well as after recovery showing that patients during and after acute infection have reduced DLCO (Núñez-Fernández, et al., 2023; Matheson, et al., 2023; Thomas, et al., 2021). Consistently low DLCO has been documented in patients diagnosed with LC up to 12 months after acute infection (Kang, et al., 2024). The inclusion of DLNO assessment allows for the determinants of pulmonary capillary blood volume (Vc) and alveolar-capillary membrane conductance (DM) (Zavorsky, et al., 2008). Understanding factor(s) responsible for the decrease in DLCO is key in understanding and developing treatment plans for patients with Long COVID. This study hypothesized that those diagnosed with long COVID would have decreased DLCO and DLNO, primarily due to decreases in Vc.

## 2 Methods

### 2.1 Study design

Subjects completed two visits on subsequent days. During visit 1, patients completed a series of pulmonary testing. These tests included lung ultrasound, assessment of fractional exhaled nitric oxide (FeNO), assessment of DLCO and DLNO, and basic spirometry testing. This testing was completed in the above listed order intentionally to prevent influence from the previous test on the results. Participants were also asked to complete multiple questionnaires including the St George’s respiratory questionnaire, short form health survey (SF-36), composite autonomic symptom score 31 questionnaire (COMP31) and the MESA COVID-19 questionnaire. Visit 2 included a chest computed tomography exam (CT). Subjects were assigned to either the non-symptomatic subject group (NS) or the Long COVID group (LC) based on their self-reported symptoms following their initial COVID-19 infection. All participants were at least 28 days out from their positive COVID-19 test date. Those within the LC group were required to be experiencing symptoms beyond 3 months past their initial infection date. Patients were confirmed to be NS or LC at the 6 months follow up visit. This study was conducted in accordance with the Mayo Clinic Institutional Review Board (IRB#21-001028). All participants provided written informed consent prior to study participation.

### 2.2 Subjects

Eighty individuals with previous COVID-19 infections were consecutively recruited for this study. Seventy-four subjects were used for analysis due to six subjects withdrawing from the study prior to completion of study participation. Subjects were required to have a confirmed positive COVID-19 test prior to enrollment. Subjects were required to be over the age of 18 and not currently pregnant. Half of the participants had no lingering symptoms following their initial infection with COVID-19. The other half continued to have persistent symptoms greater than 3 months after their initial infection date. These symptoms can include but are not limited to coughing, dyspnea, fatigue, cognitive impairment, exercise intolerance, orthostatic intolerance, chest pain and palpitations. The subjects that qualified for the LC group self-reported at least one of the previously listed symptoms to qualify for participation as part of the LC group. Subjects were excluded if they had a history of seizure disorders, cognitive disorders, or major limitations to exercise. Additionally, patients who had pacemakers or other implantable devices were also excluded due to limitations of interpretation of CT imaging.

### 2.3 Assessment of lung ultrasound

Lung ultrasound was performed by a trained study team member utilizing the Lumify handheld ultrasound device 2-D scanner at a frequency between 1.6 and 5 MHz (Phillips™). The 28-window technique was used that was previously described by Picano et al. (2006). This technique aims to evaluate the presence of B-lines or comet tail artifact. Comet tails have been shown in critical care settings to have high sensitivity and specificity in diagnosing patients with pulmonary edema (Volpicelli et al., 2006). Therefore, was utilized in this study as an evaluation of possible extravascular lung water.

### 2.4 Measurement of FeNO

Participants were evaluated for fractional exhaled nitric oxide utilizing the FeNO+ device (MedGraphics, St. Paul, MN, United States). This maneuver starts with the subject off the mouthpiece to exhale to residual volume. At a the end of this exhale, the subject is instructed to go onto the mouthpiece while wearing nose clips and take a full inhale. The device provides real time pressure measurements to show when the subject is at complete inhale at which point the subject will exhale at a rate of 50 mL/s until the device has received a full sample. The measurement of exhaled nitric oxide has been shown to be a good marker for airway inflammation, especially in those experiencing asthma related eosinophilic mediated inflammation (Barnes et al., 2010; Taylor, 2006).

### 2.5 Collection and evaluation of DLCO and DLNO

All participants were assessed for DLCO and DLNO simultaneously utilizing the Hypair device (MedGraphics, St. Paul, MN, United States). A 10-L Douglas bag was filled with 0.3% carbon monoxide, 0.7% acetylene, 10% helium, 21% oxygen, 40 PPM nitric oxide and the remaining was filled with nitrogen as previously described by Jorgensen et al. (2018). The bag was filled with 1.0 L more than vital capacity to give adequate gas without the possibility of bag collapse during inhalation. The nitric oxide was added to the bag immediately before testing. The subject was instructed to breathe room air on the mouthpiece normally for at least 4 tidal breaths, followed by a complete exhale to residual volume. At this point, a study team member initiates the switching valve to move the subject’s breathing to the Douglas bag where they take a complete inhale as quickly as possible, with at least 90% of vital capacity being reached within 4 s. The subject is coached to hold the breath for 4 s, before being instructed to exhale quickly. This maneuver was repeated three times dependent on data variability. As per American Thoracic Society standards, two measurements within 10% of the average value were reported (Graham et al., 2017). There was a minimum of 4 min in between tests for washout. Predicted values for DLCO and DLNO were derived using the reference equations of Zavorsky et al. (2008) as implemented by the HypAir system (Medisoft, Sorinnes, Belgium).

Carbon monoxide has a greater affinity for hemoglobin than oxygen, therefore it binds to hemoglobin faster and the rate of disappearance can be measured to assess how well gases are moved from the alveoli to the capillaries. Nitric oxide diffuses across the alveolar-capillary membrane faster than CO. Therefore, the differences in the rate of disappearance of carbon monoxide and nitric oxide can provide greater detail for measurements of membrane conductance (Dm) and pulmonary capillary blood volume (Vc). Reaction rate of nitric oxide with hemoglobin (ΘNO) was considered finite based on the calculations of the system used. Helium gas is used as a tracer as it is not conducted across the membrane and allows for the measurement of alveolar volume (VA) (Wheatley-Guy, 2020).

### 2.6 Gas exchange based surrogate of pulmonary vascular capacitance (GxCap)

Pulmonary vascular capacitance is the ability of the pulmonary vessels to store blood volume without significant increases in pressure. This measurement reflects the vessel’s ability to appropriately distend. This was calculated via indirect methods by end tidal CO_2_ (PETCO_2_) multiplied by O_2_ pulse, where O_2_ pulse is calculated by absolute VO_2_ divided by heart rate. VO_2_ and PETCO_2_ were measured using the Breeze Ultima system (MedGraphics, St. Paul, MN, United States) and heart rate was measured using the Maximal Radical-7 Pulse Oximeter (Masimo Corporation, Irvine, CA, United States). These non-invasive measures, referred to as GxCap, serve as a surrogate of pulmonary vascular capacitance as defined in previous studies (Wheatley-Guy et al., 2020; Taylor et al., 2013).

### 2.7 Spirometry

Basic spirometry was performed per ATS/ERS standards (Graham et al., 2017), which included slow vital capacity (SVC) and forced vital capacity (FVC) maneuver. Forced vital capacity (FVC) maneuver to identify vital capacity (VC) and obstructive disease (Stanojevic et al., 2022). Predicted spirometry values were based on the Global Lung Initiative (2012) reference equations using the Ultima system (MGC Diagnostics, St. Paul MN, United States) (Quanjer et al., 2012).

### 2.8 Thoracic computed tomography

Thoracic computed tomography (CT) scans were performed clinically by experienced radiology technologists, following standard chest CT guidelines. Chest CT was performed according to previously published methods by Wheatley-Guy et al. (2020). Scans were quantitatively assessed with the use of Matlab active contour algorithms (Mathworks, Natick, MA, United States) where lung tissue was segmented from surrounding tissue. Fluid content (FC%) was calculated as a percentage of mean lung density using the equation: FC% = (mean lung density + 1000)/10. This equation has been validated in previous studies (Simon, 2000; Kato et al., 1996; Lange and Schuster, 1999). Using FC% allows for assessment of lung fluid as a percentage of the total lung that is wet (Wheatley-Guy et al., 2020).

### 2.9 Evaluation of questionnaires

All participants completed four different questionnaires including the St. George’s respiratory questionnaire (SGRQ), short form health survey (SF-36), compass 31 questionnaire (COMP31) and the MESA COVID-19 questionnaire. The SGRQ evaluated subjects for symptoms related to pulmonary changes including dyspnea, coughing, and wheezing, with scores that range from 0, indicating absence of symptoms, to 100, indicating severe symptoms. SF-36 was selected to assess overall wellbeing with a higher score being a higher perception of general wellbeing. The results are divided evenly between physical and mental health domains, with the total score being on a scale of 0–100, with 100 indicating excellent health. Similarly, COMP31 gives a score for subject’s severity of dysautonomia related symptoms such as orthostatic intolerance, light sensitivity, and gastrointestinal symptoms. This score also ranges from 0 to 100, with 100 indicating severe autonomic symptoms. Finally, the MESA COVID-19 questionnaire was administered to better understand date of initial infection, what course of treatment was used and vaccination status.

### 2.10 Statistical analysis

All statistical analyses were performed using SPSS version 28.0. Data were assessed for normality using visual inspection of histograms and boxplots. Descriptive statistics are reported as mean ± standard deviation. Group comparisons were conducted using independent sample t-tests. A two-tailed p-value of <0.05 was considered statistically significant. Adjustment of potential confounders was done utilizing ANCOVA to adjust for age, BMI and sex.

## 3 Results

Participants included 74 subjects (23 male, 51 female) were recruited to participate in this study. Demographic information can be seen in Table 1.

**TABLE 1:** Demographics.

	All (n = 74)	Long COVID (n = 37)	Non-symptomatic (n = 37)	p-value
Gender	51 female (68.9%) 23 male (31.1%)	26 female (70.2%) 11 male (29.7%)	25 female (67.6%) 12 male (32.4%)	0.801
Age (years)	42.2 ± 15.5	47.3 ± 14.8	36.9 ± 14.3	0.010^a^
Height (cm)	168.9 ± 8.0	168.2 ± 6.5	169.6 ± 9.1	0.395
Weight (kg)	78.6 ± 19.5	80.7 ± 21.3	76.5 ± 17.2	0.539
BMI (kg/m^2^)	27.5 ± 6.2	28.5 ± 7.3	26.4 ± 4.6	0.248
Hemoglobin (g/dL)	13.89 ± 1.21	13.92 ± 0.94	13.86 ± 1.4	0.833

^a^ Denotes significance between groups set at p < 0.05.

The LC group had significantly lower DLCO and DLNO compared to the NS group (Table 2). The difference was not explained by the difference in age between the groups, as DLCO and DLNO percent of predicted was also lower between groups (Table 2). Further review of the components of DLCO, pulmonary capillary blood volume (Vc) was significantly different between groups with LC group being lower than the NS group (Table 2). Alveolar-capillary membrane conductance (Dm) was not significantly different between groups (Table 2). Alveolar volume (VA) was significantly different between groups (Table 2). The diffusion capacity of carbon monoxide corrected for alveolar volume (DLCO/VA) was not significantly different between groups (Table 2). The functional unit of gas exchange (Dm/Vc) was not significantly different between groups (Table 2). DLCO, DLNO and Vc were no longer found to differ significantly between groups after adjusting for age, sex, and BMI (p = 0.273, p = 0.135, p = 0.192). Further between group analysis revealed that only in females, not in males, was DLNO and Vc significantly lower in those with LC (p = 0.022 and p = 0.018 for DLCO and Vc respectively in females, p > 0.05 for both in males. The LC group had a significantly lower absolute and percentage of predicted forced vital capacity (FVC) when compared to the NS group, respectively (Table 2). Absolute and percentage of predicted slow vital capacity (SVC) were significantly lower between groups (Table 2).

**TABLE 2:** Summary and comparison of pulmonary and inflammatory metrics between LC and HS participants.

	All (n = 74)	Long COVID (n = 37)	Non-symptomatic (n = 37)	p-value
DLCO abs (mL/min/mmHg)	23.7 ± 7.5	21.4 ± 6.5	26.1 ± 8.3	0.015^a^
DLCO % pred	80.6 ± 20.1	77.1 ± 16.6	87.7 ± 19.8	0.029^a^
DLNO abs (mL/min/mmHg)	137.2 ± 35.9	125.6 ± 32.3	144.0 ± 35.5	0.009^a^
DLNO % pred	96.4 ± 20.2	93.2 ± 15.9	100.5 ± 17.2	0.024^a^
DLNO/DLCO	5.89 ± 0.62	6.01 ± 0.63	5.57 ± 0.47	0.122
Dm (mL/min/mmHg)	216.1 ± 95.6	208.6 ± 97.3	187.3 ± 47.9	0.540
Vc (mL)	49.2 ± 16.6	43.4 ± 13.9	56.4 ± 18.6	0.003^a^
VA (mL)	5.57 ± 1.15	5.06 ± 1.17	5.95 ± 1.16	0.004^a^
DLCO/VA	4.34 ± 0.79	4.31 ± 0.88	4.27 ± 0.90	0.443
DM/Vc	4.63 ± 2.52	4.47 ± 2.17	4.74 ± 2.79	0.871
GxCap	14.67 ± 4.93	13.52 ± 4.88	15.73 ± 4.66	0.060
FVC (L)	4.02 ± 1.03	3.64 ± 0.91	4.38 ± 1.0	0.001^a^
FVC % pred	100.9 ± 15.6	96.03 ± 105.6	105.6 ± 13.1	0.010^a^
SVC (L)	3.93 ± 0.99	3.55 ± 0.89	4.27 ± 0.96	0.001^a^
SVC % pred	98.4 ± 15.1	93.5 ± 15.7	102.9 ± 13.1	0.012^a^
FeNO (ppb)	12.6 ± 8.5	13.8 ± 10.3	11.5 ± 6.1	0.337
Comet tails	3 ± 4	3 ± 3	3 ± 4	0.298
Fluid content (FC%)	14.63 ± 2.79	14.40 ± 2.41	14.85 ± 3.04	0.525
VO2 peak (mL/kg/min)	24.1 ± 9.0	21.3 ± 8.7	27.1 ± 8.5	0.600

^a^ Denotes significance between groups set at p < 0.05.

^b^ ΘNO, was considered finite for these calculations.

Abbreviations: DLCO, diffusion capacity of the lungs for carbon monoxide (data corrected for hemoglobin); DLNO, diffusion capacity of the lungs for nitric oxide; Dm, alveolar-capillary membrane conductance; Vc, pulmonary capillary blood volume; VA, alveolar volume; GxCap, gas exchange based surrogate of pulmonary vascular capacitance; FVC, forced vital capacity; SVC, slow vital capacity; FeNO, fractional exhaled nitric oxide.

**FIGURE 1:**
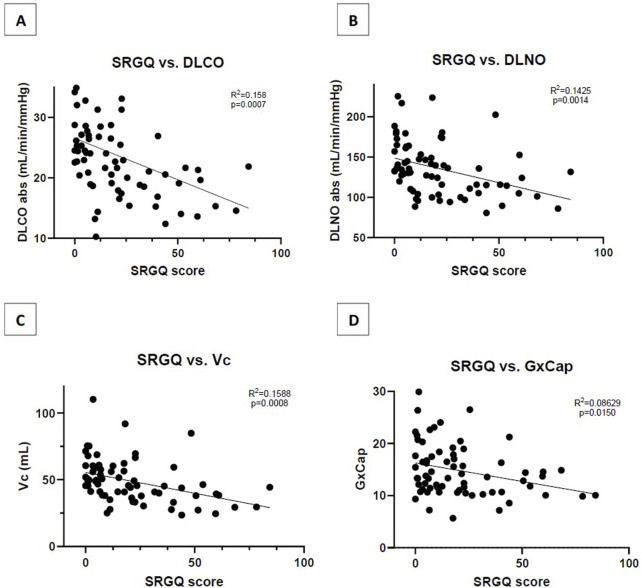
**(A)** Correlation of SGRQ score to absolute DLCO measurement **(B)** Correlation of SGRQ score to absolute DLNO measurement; **(C)** Correlation of SGRQ score to Vc measurement; **(D)** Correlation of SGRQ score to resting GxCap measurement. SGRQ, St. George respiratory questionnaire; DLCO, diffusion capacity of the lungs for carbon monoxide; DLNO, diffusion capacity of the lungs for nitric oxide; Vc, pulmonary capillary blood volume; GxCap, Gas exchange based surrogate of pulmonary vascular capacitance.

The LC group scored significantly higher on average for the SGRQ at 34.06 ± 21.5 versus the NS group that score an average of 8.46 ± 8.6 (p < 0.001). SGRQ responses showed that a reduction in DLCO is negatively related to symptoms severity based on the SGRQ score (*R* ^2^ = 0.169, p = 0.0156) (Figure 1A). DLNO testing revealed a similar negative relationship of SGRQ score to DLNO score (*R* ^2^ = 0.093, p = 0.002) (Figure 1B). A negative relationship of SGRQ score to Vc was also observed (*R* ^2^ = 0.1588, p < 0.001) (Figure 1C). For the COMP31, the LC group scored significantly higher than the NS group at 38.3 ± 17.1 and 13.8 ± 8.6, respectively (p < 0.001). On the SF36 physical health component, the LC group scored significantly lower than the NS group at 39.53 ± 12.9 and 52.46 ± 9.2, respectively (p < 0.001). The second component of the score is mental health where the LC group scored 45.63 ± 9.8 and the NS group scored significantly higher at 52.5 ± 9.2 (p < 0.001).

There was no significant difference between groups for comet tail assessment, exhaled nitric oxide, or CT data fluid content percentage (Table 2).

## 4 Discussion

Data from this study adds to the body of work surrounding DLCO which remains decreased in patient’s diagnosed with long COVID. Additionally, this study supports the literature that DLNO is also decreased among these patient’s providing a more specific assessment of where the defect in diffusion capacity of the lungs is occurring (Agostoni et al., 2024). This study demonstrated that the added value of including DLNO testing within the same maneuver has shown the additional information of Vc being decreased in patients with LC and the function unit of gas exchange (Dm/Vc) being similar among both groups.

A decreased Vc indicates that there is not enough blood available for gas exchange to occur at the alveolar level. Previous literature would support that this is a blood flow problem possibly due to microthrombi occurring in the smaller vessels (Nicolai et al., 2023). However, the findings of this study show a significantly lower alveolar volume (VA) in the LC group versus the NS group. This would suggest that the decrease in Vc could be due to a decrease in VA, as the measurement for Vc indirectly relies on volume of gas in the alveoli to get an accurate measurement (Hughes and Pride, 2012).

VA may be the reason that DLCO, DLNO and Vc are all reduced, because these measurements can only account for the lung surface area that is ventilated. A lower lung volume, concluded by lower VA, could be the reason for the decrease in all these values as there is not enough gas available for appropriate exchange to occur. This is further supported by the lack of difference in DM/Vc between groups. DM/Vc is theoretically the functional unit of gas exchange, this being unchanged shows that the individual units themselves are still efficient (Agostoni et al., 2006; Wheatley et al., 2010; Stewart et al., 2024; Kim et al., 2023). This further supports that the smaller lung volume that LC subjects have could be the primary reason these values are decreased. Additionally, when correcting DLCO to VA (DLCO/VA), there is no significant difference between groups (p = 0.443). The lack of change in DLCO/VA combined with the significant differences in absolute DLCO and VA independent of each other, suggesting that the changes in DLCO and VA are proportionately decreased. Further supporting the change in DLCO is being driven by the change in VA. This becomes evident when considering Equation 1 used to calculate DLCO (Hughes and Pride, 2012).

$$DLCO=\frac{\left(V_{A}\cdot K_{CO}\right)}{{Pb}^{*}}$$ (1)

Where:

- K_CO_ is the carbon monoxide transfer coefficient
- Pb* is the alveolar CO uptake per minute per mmHg P_CO_ (McCormack, 2020)

This is further reinforced by the below average pulmonary function testing values including FVC absolute, FVC %pred, SVC absolute and SVC %pred.

Supposing the above statements are true, it can also be presumed that this defect would prompt an issue with ventilation to perfusion matching (V/Q) (Tipre et al., 2022). Previous studies have concluded that the utilization of V/Q single photon emission computed tomography (SPECT) imaging done in patients with previous COVID infections indicated that most patients, regardless of severity of acute infection, experience V/Q defects, with 75% of the study population experiencing a reverse mismatch V defect (Katzenstein et al., 2022). The findings of this study support the evidence of a reverse mismatched V defect, indicating that a ventilation defect is more abnormal than a perfusion defect in patients experiencing long COVID. This is further strengthened as VA, FVC and SVC were all significantly reduced within the LC group. One might consider that extravascular lung water could be the reasoning for a decrease in diffusion. However, this study showed no differences between groups for lung fluid, as assessed by chest CT, and no differences in DM/Vc point to differences in air volume to be the primary reason for decreased DLCO.

Since the severity of long COVID symptoms appears to be independent of the severity of the initial infection, this finding may reflect a characteristic of the acute phase that helps identify individuals at higher risk for developing LC. The work by Katzenstein et al. (2022) also performed DLCO to compare to VQ SPECT findings revealing that there was a statistically significant relationship of DLCO and V/Q defects, although the mismatched V defect was not significant. This imaging technique is expensive, time consuming and is not recommended for all patients. Further research into the comparison of VQ SPECT imaging to DLCO/DLNO testing is warranted for a greater physiological understanding.

Decreased pulmonary vascular compliance could also drive a low Vc. The inability to appropriately recruit or distend capillaries would lead to decreased volume of blood available for perfusion. Perhaps dysfunction within the endothelial cells, indirectly activated through the inflammatory immune response initiated by infection of the virus, lead to a chronically inflammatory environment. This chronic inflammation can lead to poorer systemic vascular function and increased stiffness of the arteries (Ratchford, et al., 2021; Szeghy, et al., 2022). This study reported pulmonary compliance (GxCap) at rest. Although these findings were not significant between groups, they were significant when compared to score on the SGRQ (p = 0.015) (Figure 1D). This supports that there is loss of compliance at rest, dependent on continued burden of respiratory symptoms.

FeNO is a marker that has been reported as a measure of airway inflammation (Ragnoli et al., 2023). This increase in inflammation is further supported by the negative correlation of FENO and Dm/Vc (p = 0.007). Although inflammation is typically thought to impair membrane functionality more than capillary volume, FENO showed no correlation to Dm or to Vc independently, but only with the ratio. This suggests that this abnormality is not being driven by the absolute ability to diffuse or perfuse but rather the mismatch between membrane and capillary function. Such an imbalance could be due to a persistent dysregulation of the airway inflammatory response.

Interestingly, DLCO is still considered normal in many of the LC patients in our study, with normal DLCO values being >75% of predicted (Modi and Cascella, 2020). Perhaps this is why patients with LC tend to appear normal on pulmonary function testing but still have unexplained symptoms. Although this study does not have subject data prior to COVID infections, it would be reasonable to suspect that even a low normal DLCO value could be a significant decline for some subjects leading them to feel symptomatic. This decline to low normal DLCO and DLNO data can be supported with results from the questionnaires collected.

SGRQ was given to assess pulmonary related symptoms and was shown to have a significant negative relationship with DLCO, DLNO, Vc and GxCap. This finding demonstrates the utility of SGRQ in assessing current conditions and improvements. The questionnaire being simple, cost effective, and non-invasive can serve as a preliminary evaluation tool. A patient that scores high on the SGRQ would warrant further evaluation with DLCO/DLNO testing for more details on physiologic insights.

## 5 Limitations

This study recognizes that pulmonary function values prior to infection were not available, and it is therefore uncertain if the results of the pulmonary function testing would highlight differences in susceptibility to developing LC versus a decline in values caused by acute infection that later developed into LC. Furthermore, this study population had a very large range of LC symptom burden, spanning from mild to life debilitating and likewise a large range of treatment methods dependent on severity of acute infection, spanning from over-the-counter medications to hospitalization with mechanical ventilation. The study had very limited exclusion criteria and as such did not account for comorbidities in the subject group. When evaluating for covariates of age, sex and BMI, there was no longer a significant difference between groups. This analysis revealed that sex was a primary driver of differences in diffusion capacity and Vc, with females with LC showing significantly lower DLNO and Vc compared to their non-LC counterparts. Where as in males, there was no difference based on disease status. Nevertheless, unadjusted analyses indicate that patients with LC tend to have lower diffusion capacity compared with controls that warrant careful consideration alongside these known confounders. Due to influence of covariates on the findings, particularly sex, and our small sample size, there is limited statistical power and adjustments for all possible confounders were limited. Future research should address these limitations by enrolling a large sample size to allow for confirmation of our findings and evaluation of other confounders.

## 6 Conclusion

Understanding the physiological differences in patients with LC is essential for effective treatment and management of this patient population. This study has shown that the addition of DLNO testing has value to highlight where the impairments in gas exchange are occurring. Given results in previous research, the reduction in Vc observed in these patients is not unexpected. However, this might not only be related to the hypercoagulability state that these patients are in, but rather a decrease in VA that is decreasing DLCO and DLNO. Moreover, the relationship of DLCO and DLNO to pulmonary questionnaires show that the SGRQ score is a good predictor of the decline in DLCO and DLNO measurements and can be used to monitor and manage Long COVID. Additionally, GxCap tends to be lower in those within the LC group, especially when compared with SGRQ scores, suggesting decreased compliance. In conclusion, the addition of DLNO with traditional DLCO testing provides a more comprehensive approach to understanding the ongoing pulmonary symptoms in patients with Long COVID.

## Data Availability

The raw data supporting the conclusions of this article will be made available by the authors, without undue reservation.
